# 20 °C—A Short History of the Standard Reference Temperature for Industrial Dimensional Measurements

**DOI:** 10.6028/jres.112.001

**Published:** 2007-02-01

**Authors:** Ted Doiron

**Affiliations:** Precision Engineering Division National Institute of Standards and Technology, 100 Bureau Drive, Gaithersburg, MD 20899-8211

**Keywords:** dimensional metrology, gage blocks, gauge blocks, reference temperature, C. E. Johansson, S. W. Stratton

## Abstract

One of the basic principles of dimensional metrology is that a part dimension changes with temperature because of thermal expansion. Since 1931 industrial lengths have been defined as the size at 20 °C. This paper discusses the variety of standard temperatures that were in use before that date, the efforts of C.E. Johansson to meet these variations, and the effort by the National Bureau of Standards to bring the United States to the eventual world standard.

## 1. Introduction

While most dimensional metrologists know that the reference temperature for dimensional measurements is 20 °C,^1^ very few know how or why that temperature was chosen. Many people have thought it was, in some sense, arbitrary. In actuality, the decision was the result of 20 years of thought, discussion, and negotiations that resulted in the International Committee for Weights and Measures (CIPM)^2^ unanimous adoption of 20 °C as the reference temperature on April 15, 1931.

In cleaning up my office I found a pack of correspondence and documents that shed considerable light on how this decision was made by the Bureau of Standards, propagated through U.S. industry, and finally brought to closure at CIPM. It is an interesting piece of history that shows the high level of technical sophistication of metrologists of 100 years ago, as well as the deep commitment of the Bureau’s first Director, Dr. S. W. Stratton, to internationally based standards of metrology [1].

## 2. The Meter

The U.S. participation in the metric system began much earlier than most people realize, being one of the original signatories of the Convention of the Metre in 1875 which set up the International Committees and the International Bureau of Weights and Measures in Paris. The U.S. was also an early advocate of expanding the role of the Convention beyond its original scope of mass and length to cover a much wider range of measurements. The amendment of the original Convention was ratified in 1921, and since that time CIPM has become the primary authority for nearly all physical units.

The original Convention chose to base length on the French meter bar, which was an end standard. This end standard was replaced with a line scale, and then further replaced in 1889 with the Platinum-Iridium Meter of the Archives that was the standard until replaced with the wavelength of light in 1960. Thus, until 1960 the meter was defined as the distance between two lines on the Meter of the Archives at the temperature of the melting point of ice when supported by two cylinders of diameter at least 1 cm placed symmetrically under the bar and 571 mm apart.

The choice of the melting point of ice seems, today, like a very awkward choice for the reference temperature. It is a difficult temperature at which to actually make measurements, both because of human comfort and the fact that the dew point is generally above this temperature, and some sort of humidity control would be needed. Temperature was, and still is, a major complicating factor in length measurement. Because all materials change size with changing temperature, in order to define the length “1 meter” with a physical object we must also set the temperature for which it is “1 meter” long.

The standard way to compare length standards was to have two microscopes, one focused on each line of the segment of the length scale of interest. Using the microscopes, two lengths could be compared to very high precision. The accuracy of the measurement uncertainty depends on the uncertainty in the temperature of the bar during the measurement. Thermometry in the late 19th century was, unfortunately, not nearly as accurate as was needed to support the level of accuracy of length comparisons. The best characterized temperatures at the time were the melting point of ice and boiling point of water. The melting point was not only the more practical of the two; the melting point temperature is 3700 times less dependent on the atmospheric pressure, and thus much more reproducible. The choice of the melting point of ice was the only acceptable choice for the standard temperature for the meter at the time.

## 3. Reference Temperature for Industrial Measurements

This choice did, however, present a problem for industrial measurements which are almost never made at such a low temperature. The basic problem is that if the standard reference temperature is 0 °C, two mating parts of different materials, say steel and brass, will actually be their nominal size at 0 °C. If the parts are, instead, assembled at 20 °C, the parts will grow by their coefficients of thermal expansion (CTEs) times the temperature difference from the reference temperature. Since the CTE of steel is about 12 × 10^−6^/°C and brass is about 24 × 10^−6^/°C, the brass part will expand much more than the steel part and assembly may not be possible. Since parts are assembled, on average, at around room temperature it would seem practical to have the part length measurement refer to the assembly temperature as closely as possible.

The obvious way to overcome this problem would be to take some temperature near room temperature, say 20 °C, as the reference temperature for industrial measurements and then make a reference bar which would have the same length at 20 °C as the International Prototype Meter has at 0 °C. To accomplish this we need to know the thermal expansion of either the International Prototype Meter or the new standard between 0 °C and 20 °C. To use the International Proto-type Meter was not a reasonable choice because to preserve its length it was used as little as possible, as shown in the BIPM response to Dr. Stratton of the Bureau of Standards. Dr. Stratton wrote to ask if the U.S. could send its meter to BIPM for comparison to the International Prototype Meter. Dr. Guillaume, Director of BIPM wrote back (Bureau translation) [2]:

But comparisons cannot be made with International Prototype. The International Prototype Meter as well as the kilogram, and their certificates, are shut up in a depository, which is under the charge of the International Committee, and closed by three locks, one key of which is in my hands, the second is deposited in the Archives of France, and the third is in possession of the President of the Committee, Prof. Foerster at Berlin. The depository which is a deep cave under our laboratory, is inaccessible to me as well as to all the world. It cannot be opened and much more the prototype can not be taken out except by a decision of the Committee in session.

Thus, the CTE studies had to be made on the replica meters made for routine work at BIPM and for the members of the Commission. These studies were performed, off and on, for the next 30 years at BIPM as well as other countries National Metrology Institutes (NMIs). As late as the 7th International Conference in 1927 there were still major publications on this subject. The work was slow for both technical and bureaucratic reasons. The thermal expansion coefficient was needed to very high accuracy from the melting point of water up to 20 °C, which is a difficult task made more difficult because at a time the temperature scale itself was under serious study. Also, since the International Prototype is not generally available, the studies focused on the 29 nearly identical copies that were made at the same time out of the same batch of the Platinum-Iridium alloy. These copies were distributed to the members of the Convention of the Meter; meter bar No. 27 was the legal standard for length in the United States until the redefinition in terms of the wavelength of light in 1960. Simply getting the bars back to BIPM for measurements was time consuming. Efforts began in 1921 and continued for 15 years [3].

From a modern perspective, the basic question is that of uncertainty. Since the meter was defined as the distance between two lines of the International Prototype when held at 0 °C, a second meter bar, even if compared directly to the International Prototype at 0 °C will have a larger uncertainty when used at 20 °C: larger by the uncertainty of the change in the length of the bar when heated. The size of the uncertainty depends on the knowledge of the CTE of the gage and the accuracy of the temperature measurement. For ordinary gages measured in a lab environment, the knowledge of the CTE of the gage and the accuracy of the thermometers would add considerable uncertainty to the gage at 20 °C.

However, for one specific gage these uncertainties could be minimized. In particular, at a National Measurement Institute the uncertainty could be reduced to a level that would be negligible for industrial measurements. We will see later that C. E. Johansson, inventor of the gage block, had done exactly this for his own use in his factory. Unfortunately, in the early years of the 20th century the concept of uncertainty was still in its infancy, and most of the members of CGPM and scientists of the BIPM seem to have been swayed more by the philosophical implications than the practical merits of 20 °C.

The lines were drawn on this question fairly early, at least in the United States. In the following exchange of letters Dr. Guillaume, Director of BIPM, inquires about a number of standards issues related to the work of the Bureau of Standards [4].

I had the pleasure on June 22 of this year to write you on the following subjects, (1) hydrometers, (2) thermometers, (3) end standards, (4) question of the carat, (5) units of cold, (6) China, (7) invar tubes.

My letter remaining without reply, I fear that it has not been received, and I repeat the essential points.

…

3. For the end standards, it had to do especially with the reference temperature of 0 °C and to see how it might be received by the American industries.

The reply from Dr. Stratton shows that the question was, at least to him, already settled [5].

3. We find in this country a strong objection, even among scientific men, to the use of the temperature 0 °C as the standard for practical purposes, and we are of the opinion that it would retard the use of the metric system in this country to insist upon all measuring apparatus being correct at this low temperature. It seems to me that it would be far better to have the standards both of length and capacity agree or nearly agree at the temperature at which they are commonly used, say 20 °C, than to have them agree at 0 °C, at which temperature they are rarely used.

For example, to have brass, platinum, steel, and other length measures with widely different expansions correct at 0 °C instead of at ordinary temperatures would not appeal to most users of such measures. The tapes used by our Coast and Geodetic Survey are all made so as to be approximately correct at 20 °C. The same is true of all polariscopic apparatus and of the volumetric apparatus used by chemists. We are strongly of the opinion that the temperature of 20 °C should be selected as the temperature at which all working apparatus should be standard.

Despite the U.S. stance, the equally strong stance of Great Britain for 62 °F, and the fact that almost no one outside of France used 0 °C as the standard reference temperature, in 1915 the 5th International Conference passed the following resolution [6].

DECLARATION RELATIVE TO END MEASURES.

Translation.

Considering the precision of adjustment has become an indispensable factor, both for the proper functioning of machines and for manufacturing in series, an essential element in all industrial construction,

Considering the necessity of referring to a single scale the materialization of dimensions expressed numerically by the plan carried out,

Considering that this condition cannot be realized except by the choice of a single temperature of adjustment at which industrial standards shall actually represent their numerical value,

Considering, the International Committee, in its Session of 1909, had already sanctioned for adjustments the temperature of melting ice by the adoption of which the joint action of several official bureaus had already begun the desired unification.

The Conference declares:

1. Approval of the fixing of the temperature of melting ice as the adjustment temperature at which end standards intended for control of industrial manufacturing should possess their nominal value.

2. To invite the International Committee to undertake all the necessary work which will assure the highest perfection in construction, determination and use of end standards.

This ironic Declaration seems to have little effect. As an example, in 1915 Dr. Stratton received a letter from Dr. R. T. Glazebrook of the National Physical Laboratory in Great Britain along with a very detailed analysis by M. J. E. Sears, Jr., the Deputy Warden of Standards in Great Britain. In this work Mr. Sears details the problems with the 0 °C reference temperature for industrial measurements and makes the case for the British standard temperature of 62 °F (16.67 °C).

Answering for Dr. Stratton was E. A. Rosa^3^ [7]:

We have found some discrepancies in the apparent temperatures of standardization of other metric apparatus o the part of the manufacturers, due largely, we believe, to lack of appreciation of the importance of care in the matter, or to lack of an understanding of the whole subject. We shall be pleased to gather more exact data as to the practice in this country with a view to recommending a definite policy and shall communicate the results to you in a later letter.

Unfortunately, the follow-up never was written and Dr. Glazebrook wrote back the next year [8]:

On June 3rd, 1915 you wrote acknowledging a letter of ours as to the standard temperature for commercial metric standards.

You stated you would write further on the return of Mr. L.A. Fischer, Chief of the Weights and Measures Division but no further letter has readied us. The matter is assuming increased importance and I should value your views greatly. Dr. Guillaume, of course, adheres to Standardization at zero centigrade.

Yours very truly,

R. T. Glazebrook.

**Fig 1:**
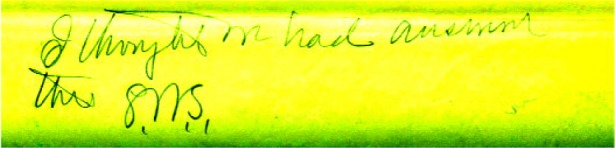
Hand written note from Dr. Stratton at bottom of letter from Dr. Glazebrook of the National Physical Laboratory (Great Britain).

At the bottom of the letter is a handwritten note from Dr. Stratton (SWS) Fig. 1.:

While there is no record of the follow up letter, mail delivery was apparently a problem. A large number of correspondences in the files are simply letters acknowledging the reception of correspondence, a habit that would not be needed if mail delivery was assured. But it is also true that the number of requests for information sent to the Bureau of Standards about the standard temperature problem was rising, and the number of Bureau staff was still quite small.

Dr. Stratton’s answer on October 16, 1916 reveals no change in his attitude about the problem [9]:

Referring to your letter of August 17, 1916, in regard to a suitable standardization temperature for commercial metric standards of length, I have to say that we have carefully read Mr. Sears’ memorandum, and while we agree with him that commercial standards of length, whether metric or Eng1ish, should be standardized at the temperature at which they are to be used, we do not concur in his opinion that the mean work-shop temperature to be se1ected should be 62 °F.

There is, at the present time, a decided tendency away from the Fahrenheit temperature scale, and we feel that the tendency should be encouraged. There is, in fact, a bill now pending in Congress by which it is hoped to abolish the Fahrenheit scale, at least from Government publications.

The temperature 20 °C is coming more and more to be accepted as the standard temperature for industrial as well as scientific operations. The sugar industry, for example, is practically on the 20 °C basis. All polariscopic tubes, flasks, etc. used in making up sugar solutions are made standard at that temperature. Very many hydrometers are standard at this temperature and the glass volumetric apparatus standardized by this bureau is on that basis and has been for the past ten years or sore. Also many of the steel tapes used in this country are standard at 20 °C.

I might add many other examples to show that 20 °C is being largely accepted as the standard temperature in scientific and technical work. Would it not, therefore, under the circumstances, be better to standardize both the English and metric commercial standards on this basis rather than that of 62 °F? 20 °C would certainly have a very great advantage over 62 °F if urged for international adoption; and from. a practical point of view it would be no more difficult to change the English commercial standard from 62 °F to 20 °C (68 °F), than to change the metric standards from 0 °C to 16.67 °C (62 °F).

Although Dr. Stratton strongly advocated 20 °C as the reference temperature, the Bureau of Standards, itself, was not completely standardized in its measurements. In response to a request from one of the Standards Committees of the American Society of Mechanical Engineers (ASME) the Director asked Paul Agnew^4^ to review all of the “Standard Temperatures” in use at the Bureau. His response to ASME, written in January of 1918 shows the span of the problem. After discussing how 20 °C, 25 °C, and 62 °C were all in use in different fields at the Bureau, the situation worldwide was even worse. He concludes with [10]:

The average laboratory temperatures in Europe are decidedly lower than in nearly the whole of the United States, and it is doubtful whether international agreement is possible on so high a temperature as 25 °C. There has been much difficulty in getting Europeans to agree even to 20 °C. Various temperatures have been suggested and used abroad, 15 °C, 16°C, 17.5 °C, 18 °C, etc., for different standardizing purposes. 15 °C was formerly the one in greatest use.

I find considerable difference of opinion among the men at the Bureau. Most of the chemists feel strongly that 20 °C is the temperature that should be used as a standard. Most of the electrical men, but not all, prefer 25 °C while those in length and volumetric measurements prefer 20 °C.

In maintaining a temperature for experimental work 25 °C is frequently much more convenient than 20 °, since it is so much easier to heat than to refrigerate. In electrical work another consideration enters. Even if one does go to the trouble to refrigerate in summer, it will frequently happen that the humidity then becomes very high, even to saturation, and insulation problems become acute.

It seems to be doubtful whether a single standard temperature of reference is possible for all the interests involved.

For industrial gages, other than tapes^5^, however, the question had been quietly settled for the U.S. during the First World War. The story of the change, basically by fiat of the Bureau of Standards, is summarized by Lewis Fisher^6^ in a letter to Paul Agnew, who was the Executive Secretary of the ASME and the American Engineering Standards Committee (precursor to the current American National Standards Institute (ANSI)) [11].

1. Referring to your communication of May 27th, subject—Reference Temperature for Steel gages—I have to state that the practice in this country is somewhat mixed, but so far as the gage work is concerned, all gages are made standard at the temperature of 68 °F or 20 °C. This was done at the beginning of the war and consequently all gages used during the war were standardized at this temperature.

2. The Johansson gages which were sold to us at about the time we adopted this temperature were standard at 66 °F. The origin of this temperature is entirely unknown to me. It seems to have been original to Mr. Johansson, never to my knowledge having been used by anyone else.

The mystery of these 66 °F blocks would not be solved for another few years.

While the Bureau was using 20 °C for nearly all length other than surveying tapes, it is not clear how well this information was disseminated to industry. As examples, the following two letters came to the Bureau from the W. & L. E. Gurley Company and the Brown and Sharpe Company, both manufacturers of precision dimensional measurement equipment sent these letters to the Bureau in 1922 [12, 13]:

Gentlemen:

Referring to your letter of the 13th instant, relative to the above subject, we wish to call your attention to the fact that while we handle Tapes only on a resale basis, we do make Yard Measures, Leveling Rods, Stadia Rods, etc., which are standard at 62 degrees F.

We are further under the impression that the standard for weights and measures is 62 degrees F. We are raising this point wondering whether there will be any confusion due to one standard for Tapes and another standard for the line in which we are interested, as noted above.

Yours Very Truly,

C. I. Day, General Manager

W. & L. E. Gurley

*My dear Mr. Bearce^7^*:

In discussing the question of temperatures at which gages should be measured, and in view of what we understand to be the present practice, of the Bureau of Standards, using 68 degrees F. as the working temperature, it would be of interest to us to know how long the Bureau has worked on the basis of this temperature, and what the reasons were for changing from 62 degrees, which we understand was used at one time.

Information along these lines would be appreciated by

Yours Truly,

Brown & Sharpe Mfg. Co.

The answer from the Bureau was consistently the same [14]:

In reply to your letter of the 13th instant, we would say that at the time of the entrance of the United States into the War, the Ordnance Department proposed to adopt 66 °F as the standard temperature for gages because at that time 66 °F was the standard temperature for Johansson gage blocks which were to be the master reference gages for all gage work. The Bureau contended that if any change was to be made from 62 °F, it should be made to 68 °F (20 °C) which is the standard temperature for the majority of physical constants and is the usual temperature for laboratory or inspection room work. Furthermore, European countries at the time were in a transition period from a standard temperature of 0 °C for length standards to 20 °C, and it was felt that an international standard temperature for length work was very desirable. We would say that at the present time both Johansson gage blocks and Pratt & Whitney gage blocks are regularly furnished in this country standard at 68 °F. 68 °F has also been adopted by the National Screw Thread Commission and by the A.S.M.E Sectional Committee on Plain Limit gages, as the standard temperature for gages and gaged products.

Fortunately, the difference between the inch defined at 62 °F and 68 °F was not a serious problem given the manufacturing tolerances of the time.

## 4. Expansion of the Scope of International Standards Work

The question of the Standard Reference Temperature was dormant for a few years after the war because of larger issues involving new fields of measurement. Even before the war, the rise in importance of electricity to industry was obvious, and the need for standardization of the units and measurements grew greatly during the war years. Dr. Stratton of the Bureau of Standards had greatly expanded the electrical capabilities during the period, and as the war ended began a campaign to enlarge the scope of the CGPM from just length and mass to all of the new fields that affected industry. The formal resolution, below, was passed at the March 18, 1919 interim meeting of the National Research Council [15].

On behalf of the Division of Physical Sciences, Leuschner reported on the desirability of enlarging the functions of the International Committee on Weights and Measures and moved: That the Division be authorized to appoint a special committee to prepare suitable recommendations in this respect.

At the first meeting of this new committee, the minutes show extensive discussion of the subject, and the decision that, since the matter was very complicated, a smaller subcommittee would be appointed to prepare recommendations for the full committee to consider. This smaller committee was made up of Dr. Joseph S. Ames of Johns Hopkins University, Professor A. A. Michelson of the University of Chicago, Dr. John A. Anderson of the Mount Wilson Observatory, and Dr. Stratton, Director of the Bureau of Standards.

This proposal was then taken through the Commerce Department, led by Herbert Hoover, to the State Department. Since the CGPM is a diplomatic organization made up of states, the U.S. representative is to be appointed by the State Department. Dr. Stratton was granted full power to sign the Convention as modified at the CGPM meeting of October 27, 1921. A large number of changes were made during this meeting of the CGPM, including the extension of the scope to electrical measurements.

After this, the efforts concerning international standardization decreased markedly. Part of the reason was economic. During the war the duties and staff of the Bureau had grown explosively, from a budget of less than $1 million and a staff of 400 in 1914, to over $3 million and 1150 staff in 1918. In the following years the decrease was nearly as fast, shrinking to 2/3 of the war year levels in 1925. Because of numerous economic factors, including a depression in 1920, this period was one of retrenchment and reorganization. Another reason was the departure of Dr. Stratton in January of 1923 to become President of M. I. T. Dr. Stratton had always had a keen interest in standardization, and was very active in all phases of both national and international standards efforts. On his leaving, Secretary Hoover appointed him to the Visiting Committee, a liaison group of prominent men (and later women) from science and industry who were to keep the Secretary of the Treasury^8^ apprised of national matters that were in the Bureau of Standards domain, and to report yearly on the work of the Bureau.

During this period, up to 1927, there was little done on the question of the reference temperature for dimensional measurements. The U.S. had been standardized at 20 °C by the Bureau of Standards, and since the CGPM Declaration of 1915 was widely ignored the issue was still a problem internationally.

## 5. Proposals to the 7th Meeting of the CGPM in 1927

In early 1927 the Bureau of Standards began to formulate a number of proposals to the 7th meeting of the CGPM, which was to be held in October 1927. The process ended up with five [16]:

1. Recommend that the Conference adopt the wavelength of the red radiation of Cadmium vapor as the fundamental standard for the wavelength of light, and indirectly for the meter. A committee was formed to look into the matter. (Prof. Kösters of Germany had found that the light from Krypton gas had better properties than Cadmium, and the group decided to make further studies of the properties of Krypton lamps. Krypton lamps were made the standard for length in 1960.)
2. Recommend that the Conference adopt the relation 1 inch equals 0.0254 meters. This was considered out of the scope of the international body since it did not involve the metric system. It was referred to the parties involved (English speaking countries) for resolution. (The value 1 in = 25.4 mm was adopted by the National Measurement Institutes of the countries that still used inches in 1959).
3. Recommend that the Conference adopt 20 °C (68 °F) as the standard temperature at which industrial standards of length shall have their correct nominal length. A committee of five members was set up to study this question and report before the first of March, 1929. The following were the members:
  Bureau of Standards, Washington;
  Laboratoir d’Essais du Conservatoire des Arts et Metiers, Paris;
  National Physical Laboratory, Teddington;
  Physikalisch-Technische Reichsanstalt, Charlottenburg;
  Director Guillaume of the International Bureau.
4. Recommend that the Conference consider the desirability of having all certificates issued by the International Bureau of Weights and Measures contain all relevant test data to permit conditions of comparison to be known and reproduced, in order that reduction to such other standard conditions as may be required by National Standardizing Laboratories may be made with the highest accuracy.
5. In view of the ever increasing demands for a knowledge of the exact lengths of the national Prototype Meter Standards, it is recommended that the Conference urge upon the International Bureau of Weights and Measures the desirability of expediting the study of the lengths and coefficients of thermal expansion of these standards, in order that any necessary changes of certificates may be authorized by the Eighth General Conference.

## 6. A U.S. Industry Consensus

Work on all of these, except the issue of the inch, were taken up very quickly. The Director of the Bureau of Standards, Dr. Burgess who succeeded Dr. Stratton in 1923, immediately began a round of correspondence with interested parties in the United States to assess the impact of the change in reference temperature. Henry Bearce, head of the Weights and Measures Section of the Bureau. prepared a list of important industry and government representatives to invite to a conference to decide the proper course for the U.S. delegates to the Conference [17]. Chosen were:

Bureau of Standards, Washington, D.C.
Gage Division, Ordnance Dept., U.S.A., Washington, D.C.
Engineering Bureau, Navy Department, Washington, D.C.
National Screw Thread Commission, Washington, D.C.
American Society of Mechanical Engineers, 29 West 39th St., New York, N.Y.
Society of Automotive Engineers, 29 West 39th St., New York, N.Y.
National Machine Tool Builders’ Association, Cincinnati, Ohio
American Railway Association, 30 Vesey St., New York City
Pratt & Whitney Co., Hartford, Conn.
Brown & Sharpe Mfg. Co., Providence, R. I.
Johansson Div., Ford Motor Co., Detroit, Mich.
Taft-Peirce Mfg. Co., Woonsocket, R. I.
Greenfield Tap & Die Corp., Greenfield, Mass.
Sheffield Mach. & Tool Co., Dayton, Ohio
Standard Gage Co., Poughkeepsie, N. Y.
The L. S. Starrett Co., Athol, Mass.
The Van Keuren Co., 12 Copeland St., Watertown, Boston 72, Mass.
Lt. Col. E.C. Peck, Room 305 Lake Erie Bank Bldg., 1612 Euclid Ave. Clev., Ohio.
S. W. S. Stratton, Pres. Mass. Inst. of Tech., Cambridge, Mass.

On April 13, 1928 Dr. Burgess sent identical letters to all of the companies and individuals on Bearce’s list. The letter said:

At the last meetings of the International Committee of Weights and Measures and the General Conference on Weights and Measures held in Parts last September and October, the question of international agreement on the standard temperature for intercomparison of industrial standards of length, such as precision gage blocks and other end standards, graduated scales, lead screws, etc., was discussed, and there was set up a committee of five members to study this question and report before the first of March, 1929. The following are the members: Bureau of Standards, Washington; Laboratoir d’Essais du Conservatoire des Arts et Metiers, Paris; National Physical Laboratory, Teddington; Physikalisch-Technische Reichsanstalt, Charlottenburg; with Director Guillaume of the International Bureau.

The American delegates supported by practica1ly all the Europeans except the British and French proposed 20 degrees C (68 degrees F), the British 62 degrees F (16 2/3 degrees C), and in France I believe both 0 degrees C (32 degrees F) and 20 degrees C (68 degrees F) are in use.

It is considered desirable to get a consensus of opinion from the engineering and industrial activities of the various countries and with this object I am cal1ing a conference at an early date at the Bureau of Standards and request your organization to designate a representative.

The early responses were both unanimous and definite that 20 °C was the proper choice.

“Our practice is invariably to give 68 degrees Fahrenheit as standard, and we have no doubt that this practice is well nigh universal in the engineering and inspection departments of American Industry.”

1928-04-16 Taft-Pierce response

“Replying to yours of the 13th, we certainly favor the use of 68 degrees Fahrenheit as a standard temperature for intercomparison of gauges, etc.”

1928-04-17 Brown & Sharpe response

“In reply to your letter of April 13th, we wish to be placed on record as favoring 68 °F. (20 °C.) as the standard temperature for intercomparison of standards of length. This temperature is one which most nearly approaches the average shop condition, and is a temperature in which production can be maintained as efficient standards.

1928-04-19 Pratt & Whitney response

Because of the swift and unanimous responses, Dr. Burgess decided that the matter would not need a conference and wrote again to see if there were any objections to settling the matter by mail. There were no objections and all of the responses, but one, were definite on the matter that 20 °C was the preferred reference temperature. The only other response was from the Society of Automotive Engineers, which suggested “rather detailed questionnaire asking specifically about present practice by individual companies, to what their practice applies, and other leading questions would be a very helpful index as to whether a general conference should not be called later on. It would also indicate what reference temperatures are most generally in use.” By the end of May, the date of this response, the question was already settled.

With the U.S. position now set by industry and government preference, the question moved to the international arena.

## 7. The French Proposals

There were two large works discussing the problem from the French perspective. One, an article in Le Genie Civil by A. Perard of the BIPM, and a second long report by M. F. Cellerier, Director of Laboratoir d’Essais du Conservatoir National des Arts et Metiers. Cellerier discusses a number of proposals and favors one by M. Gaux that is very similar to that of Perard.

The proposal, despite the strong reactions against it, is not as strange as it first appears if compared to mass metrology at the time. In mass metrology the largest environmental factor is air buoyancy. From very early times [18], mass metrologists had made allowance for this factor for industrial measurements using the concept of “apparent” or “conventional” mass. The apparent mass was the true mass with the correction for air buoyancy that would be accurate if the weight had the same density as brass (later revised to 8.0 gm/cc) and the density of air was 0.0012 gm/cc. For most metal weights, the assumed buoyancy correction was within 10 % of the true correction and for industrial purposes the system was accurate enough that further corrections were not needed. This system is still in use for most commercial weighing systems.

In analogy to “conventional” mass, the Graux [19] and Peraud [20] proposals would have effectively invented a quantity “valeur-type” which might be understood as “conventional” length. The largest environmental factor in length measurement is temperature, so the conventional length would differ from the true length (referred to the standard temperature 0 °C) by assuming a common CTE (11 × 10^−6^ / °C) and industrial reference temperature (20 °C) that would be accurate enough for much industrial measurement. Since most industrial parts are iron or steel, this method would reduce the error from thermal expansion by 90 %, which would be adequate for many industrial measurements. Unfortunately, the relative size of thermal errors in length measurements and mass measurements are quite different, and while the “conventional” mass idea is still useful today, the “conventional” length would have been inadequate for precision parts even in 1927.

The suggestion was forcefully rejected at the Bureau by a number of scientists, as in the example below:

The proposal of Perard is impractical for several reasons. First, it assumes a coefficient of expansion and employs it over a range of 20 °C whereas it is known that gage blocks have coefficients differing somewhat from block to block and differing considerably from the proposed assumed value.

In the second place an unnecessary complication is introduced. in the computation, a complication which is confusing. A quantity, termed by Perard, “valeur-type”, is introduced the physical meaning of which is not readily apparent.

1927-09-08 Judson to Burgess on Perard paper

Perard’s suggestions did not fare any better in Europe. In this excerpt from a letter from Dr. Kösters in Germany to Dr. Stratton (translated by Prof. Vogel) dated August 27, 1928 he gives the German position:

1. Adjusting temperature for industry measures.

…

For 1. they are of the opinion in Germany, that one can no longer depart from 20 °C, since 20 °C alone gives a clear and plain definition, and, one could no longer return to 0 °C without giving up the attained precision again. By Perard. a proposition for comparison is now made in an essay. The measure with the designation of “1 m,” is said. to be at 20 °C = 1 m + 11 × 20 µ, no matter of what material the measure consists. This proposition will not be accepted under any circumstances in Germany 9, since it is wholly unclear, and, departs from the ideal conception of the meter as an independent unchangeable length.

The temperature of 0 °C is also not debatable since at 0 °C one can not measure. I presuppose that you are of the same views and I figure that you cling to 20 °C = 68 °F.

In another report for the 1931 CIPM meeting, M. J. E. Sears, Jr., Deputy Warden of Standards for Great Britain, presented the case for 62 °F as a standard [21]. One interesting fact brought up by Sears is that the pressure scale, which is measured in inches or mm of mercury would be changed if the reference temperature for length were modified. New tables would be needed for mercury barometers. The final paragraph, however, reported that Great Britain and NPL would change the new standard temperature if international agreement was obtained.

## 8. C. E. Johansson Letter

One of the most important reports was from the most widely known and respected length metrologist in the world, C. E. Johansson. In 1928 Dr. Burgess, of the Bureau, asked for Mr. Johansson’s thoughts on the question of International agreement on the standard temperature for industrial length measurements. Mr. Johansson responded with a history of his efforts in standardizing length standards, as well as his philosophy of measurement and recommendations. The entire letter is presented as the appendix to this paper. Notice the pencil marks which denote changes to be made for translation into French and submission to the CGPM as a report.

**Fig. 2:**
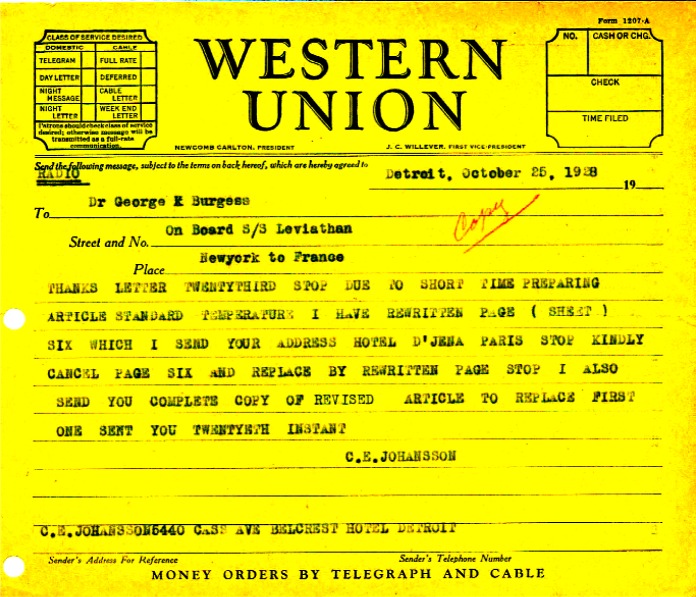
Telegram from C. E. Johansson to Dr. Burgess en route to the CGPM meeting in Paris.

The letter displays the great sophistication of Mr. Johansson as a metrologist, and shows that he had recognized the problems with the varying reference temperatures almost from the beginning of his invention of gage blocks. There are actually two letters, one was sent to Dr. Burgess and was taken to France for the meeting. Mr. Johansson discovered that he had made a mistake in his letter and sent a revised copy to the hotel in Paris where Dr. Burgess was to be staying (See Fig. 2). The section that was revised described his efforts to assign a standard reference temperature, and the specific change was to add the story of the 66 °F gage blocks sets that the Bureau received from the Ordnance Department during World War I.

Because of his continuing interest in international standards, and his standing in the world metrology community, Dr. Stratton had been retained as a U.S. official delegate to the CGPM even after he left the Bureau in 1923 and continued until his death in October, 1931. In what was probably his final act as the U.S. representative, Dr. Stratton cast his vote to adopt 20 °C as the industrial reference temperature in April, 1931 [22].

Johansson’s most interesting, and one of the earliest experiments was in 1903 when he sent a nominal 100 mm gage to BIPM and asked for the temperature at which the gage was exactly 100 mm long. His eventual answer a year later [23] was that the block was, to experimental uncertainty, exactly 100 mm at 20.63 °C. He then made a block 0.0007 mm longer, one that would be an “absolute” measuring standard of 100 mm at 20 °C. To check on his process as it evolved, in 1912 he sent four blocks 100 mm, 50 mm, and two 25 mm blocks to BIPM for calibration. At 20 °C, all were found to be their nominal size to better than 0.1 micrometer.

When Johansson began making length standards it was obvious that different customers had different reference temperatures. His solution was to buy measuring equipment and standards from different sources, measure them against his standards that had been measured at BIPM, and assign the reference temperature for each customer through his experiments. He found, for example, Brown & Shape Company in the U.S. used 62 °F, Ludwig Lowe, Berlin used 25 °C, Reinecker, Chemnitz used 14 °C, and the State Works of France 0 °C. Early sets for Japan were made for 62 °F, but after 1926 when the metric system was adopted all sets were adjusted for 20 °C. Also, from his work he found that the average temperature in laboratories and shops where his gages were used was about 20 °C, so he used that as the default unless the customer specified differently.

In another experiment in 1905, he sent a 3 inch standard to the Bureau of Standards in Washington for calibration. The Bureau issued a report giving the length of the standard at 27.2 °C. Knowing the coefficient of expansion of the block and his measurement of the block he found that the Bureau would be “nearly correct” for a reference temperature of about 66 °F. He then made blocks for the American market, primarily the War Department, that were marked as 66 °F. This odd reference temperature was not noted until the Ordnance Department began working with the Bureau of Standards during World War I. Johansson then says that he had always made metric blocks to the 20 °C reference temperature, and after his excellent agreement with BIPM in 1912 he made all inch size blocks for the American market to the 20 °C reference temperature. It does not appear that anyone in the States noticed the change.

Besides the practical advantage that most countries were using 20 °C as the reference temperature, Johansson mentions two other advantages. First, when he made gages at 62 °F the effects of the operators’ body heat affected the gages faster, reducing the amount of time available for final adjustments of length. The second was that 68 °F and 20 °C were both integers, and calculations were easier.

He finishes with a discussion of his principles for measuring. For shop inspection he advises [24]:

**V Working Temperature**

a. **Gage Work**. The temperature in the working-room, the work pieces and the gages should correspond with each other, and in accurate work, i.e., manufacturing, adjusting and calibrating of high precision gages and the like, the standard temperature + 20 °C = + 68 °F, must be held.

b. **Production Work**. The ideal condition for producing and measuring of interchangeable machine parts and the like would be to do all work and inspection at 20 °C – 68 °F, then the most severe trouble would be eliminated what measuring-values are concerned.

But when this can not be done, the next best and more possible way is to use cooling plates or other means for bringing the Work and the Gages to the same temperature. In. this case the difference in coefficient of expansion only would change the measuring-value, and in most cases it would be found to be well within the given tolerance and thus a cheap and good interchangeable product can be obtained.

This last idea is still the core idea for inspection of high precision parts, an idea that is mentioned in very few textbooks on the subject of dimensional metrology and inspection.

## 8. International Agreement of Standard Reference Temperature

The issue was finalized at the CIPM meeting on April 15, 1931. With no discussion and a voice vote, the following proposal was adopted unanimously [25]:

As the normal temperature for adjustment of industrial standards, the Committee adopts the temperature of 20 °.

The adoption of 20 °C as the reference temperature for length measurements was later incorporated into the international standards system as the first standard of the International Organization for Standardization (ISO 1) in 1951. In the United States, the National Standard ASME/ANSI Y14.5: Dimensioning and Tolerancing assigned the default temperature of 20 °C for all dimensional drawings.

There have been occasional attempts to change the reference temperature, the latest in the early 1990s [26]. These efforts have failed primarily because of the large cost of implementing the change. In 1931 the cost of the change was restricted to only a few countries and the costs were manageable; neither of these conditions holds true today. Given the very tight tolerances for modern parts and the large effect of temperature, it is unlikely that the reference temperature can be changed again.

## Footnotes

1 The original manuscripts have a variety of ways of writing the temperature units. I have changed them to the modern accepted notation for clarity.

2 The General Conference on Weights and Measures (CGPM) is the international body made up of official delegates from the countries that are signatories of the Convention of the Meter. The International Committee for Weights and Measures (CIPM) is a group of eminent metrologists that brings technical matters to the CGPM and is in charge of implementing the CGPM decisions. The International Bureau of Weights and Measure (BIPM), in Paris, holds the International Standards and coordinates the standardization of measurements at the various national measurement institutes.

3 Dr. Edward B. Rosa was hired by Dr. Stratton to head up the electrical work when the Bureau was formed in 1901. He died at his desk at the Bureau in 1921.

4 Paul Agnew joined the Electrical Section of the Bureau of Standards in 1906, and became the Executive Secretary of the American Engineering Standards Committee (forerunner to ANSI) in 1919. He served the AESC for almost 30 years. Some of his letters are as a Bureau employee, but some are on ASME letterhead where he was functioning as the Secretary to standards organizations.

5 Surveying tapes that were marked in inches were calibrated to a standard temperature of 62 °F, while those in metric units at 20 °C. When the inch was changed to be 0.0254 meters in 1959, the old surveyor inch (1 meter = 39.37 inches) was kept for surveying. Both practices continued far after the issue was settled for all other length measurements.

6 Lewis Fisher had worked in the Coast and Geodetic Survey from 1880 to the founding of the Bureau, at which time he became one of the first members of the new organization as head of Weights and Measures until his death in 1921.

7 Henry Bearce rose from an assistant in the Gage Section of Weights and Measures to Director of Weights and Measures over a career that spanned 1908–1945.

8 The original Bureau of Standards was part of the Department of the Treasury. When the Department of Labor and Commerce was formed in 1903 the Bureau was moved into the new department.

9 Underlined in the original.

## 10. Appendix

The second letter from C. E. Johansson to Dr. Burgess is presented here in its entirety. The other documents cited in this paper can be found on the Engineering Metrology Toolbox website, http://emtoolbox.nist.gov/Main/Main.asp.

## References

[b1-v112.n01.a01] 1All of the notes on Bureau staff are derived from R.C. Cochrane, Measures for Progress, National Bureau of Standards, 1966.

[b2-v112.n01.a01] 21909-11-13, Letter, Guillaume to Stratton.

[3] (1975). The International Bureau of Weights and Measures 1875–1975, translation of the BIPM Centennial Volume.

[b4-v112.n01.a01] 41909-10-21, Letter, Guillaume (BIPM) to SWS.

[b5-v112.n01.a01] 51909-11-13, Letter, Stratton to Guillaume.

[b6-v112.n01.a01] 6Bureau of Standards translation, Fifth General Conference of Weights and Measures, 1915.

[b7-v112.n01.a01] 71915-06-03, E. B. Rosa to Glazebrook.

[b8-v112.n01.a01] 81916-08-17, Dr. Glazebrook (NPL) to Stratton.

[b9-v112.n01.a01] 91916-10-16, Letter, Stratton to Dr. Glazebrook (NPL).

[b10-v112.n01.a01] 101918-01-11, Letter, Agnew to F. P. Cox, Manager of the West Lynn plant of General Electric.

[b11-v112.n01.a01] 111920-06-18, Fisher to Agnew.

[b12-v112.n01.a01] 121922-07-21, Letter, Gurley to Bureau of Standards.

[b13-v112.n01.a01] 131922-04-13, Letter, Brown & Sharpe to Bearce.

[b14-v112.n01.a01] 141922-04-26, Letter, Bearce to Brown & Sharpe.

[b15-v112.n01.a01] 15Minutes of National Research Council meeting of 1919-03-18.

[b16-v112.n01.a01] 161927-08-08, U.S. Proposals in English with markups.

[b17-v112.n01.a01] 171928-04-13, Memorandum from Bearce to Burgess.

[18] (1918). NBS Circular No. 3, Design and Test of Standards of Mass.

[b19-v112.n01.a01] 19Rapport de M. F. Cellerier in Procès-Verbaux Des Séances, Comité International des Poids et Mesures, Session de 1929.

[20] Peraud A (1927). La Température d’Ajustage des Calibres Indsutriels. Génie Civil du.

[21] Sears JJE (1931). Sur la Témperature d’Ajustage des Étalons Industriels de Longueur. Procès-Verbaux Des Séances.

[b22-v112.n01.a01] 22Procès-Verbaux Des Séances, Comité International des Poids et Mesures, Session de 1931.

[b23-v112.n01.a01] 23C. E. Johansson 1864–1943: The Master of Measurement, by Torsten K. W. Althin, published by C. E. Johansson AB, Stockholm, 1948.

[b24-v112.n01.a01] 241928-10-25 Letter C. E. Johansson to Stratton

[b25-v112.n01.a01] 25Comme température normale d’ajustage des mesures industrielles, le Comité adopte la temperature de 20 °C, from Procès-Verbaux Des Séances, Comité International des Poids et Mesures, Session de 1931, p. 63. Translation in report of the meeting prepared by Mr. Crittenden for Dr. Burgess and sent to Dr. Stratton for his approval, June 2, 1931.

[26] Blaedel K, Parsons F (1993). ISO Studying Reference Temperature Change. Quality Magazine.

